# Política pública en cuidados paliativos y sus implicaciones sobre servicios, opioides y educación en Colombia

**DOI:** 10.15649/cuidarte.2501

**Published:** 2023-09-02

**Authors:** Miguel Antonio Sánchez-Cárdenas, Laura Aguilar-Obregón, María Bernal-Tovar, Karen Gómez-Serrano, Ana Rubiano-Albarracín, Marcela Tarazona-Álvarez, Daniela Vanegas-Gutiérrez, Genny Paola Fuentes-Bermúdez

**Affiliations:** 1 Universidad El Bosque. Facultad de Enfermería. Bogotá, Colombia. Email: sanchezcmiguel@unbosque.edu.co Universidad El Bosque Universidad El Bosque Facultad de Enfermería Bogotá Colombia sanchezcmiguel@unbosque.edu.co; 2 Universidad El Bosque. Facultad de Enfermería. Bogotá, Colombia. Email: laguilaro@unbosque.edu.co Universidad El Bosque Universidad El Bosque Facultad de Enfermería Bogotá Colombia laguilaro@unbosque.edu.co; 3 Universidad El Bosque. Facultad de Enfermería. Bogotá, Colombia. Email: mbernal@unbosque.edu.co Universidad El Bosque Universidad El Bosque Facultad de Enfermería Bogotá Colombia mbernal@unbosque.edu.co; 4 Universidad El Bosque. Facultad de Enfermería. Bogotá, Colombia. Email: ktgomez@unbosque.edu.co Universidad El Bosque Universidad El Bosque Facultad de Enfermería Bogotá Colombia ktgomez@unbosque.edu.co; 5 Universidad El Bosque. Facultad de Enfermería. Bogotá, Colombia. Email: arubianoa@unbosque.edu.co Universidad El Bosque Universidad El Bosque Facultad de Enfermería Bogotá Colombia arubianoa@unbosque.edu.co; 6 Universidad El Bosque. Facultad de Enfermería. Bogotá, Colombia. Email: mtarazonaa@unbosque.edu.co Universidad El Bosque Universidad El Bosque Facultad de Enfermería Bogotá Colombia mtarazonaa@unbosque.edu.co; 7 Universidad El Bosque. Facultad de Enfermería. Bogotá, Colombia. Email: davanegas@unbosque.edu.co Universidad El Bosque Universidad El Bosque Facultad de Enfermería Bogotá Colombia davanegas@unbosque.edu.co; 8 Universidad Nacional de Colombia. Facultad de Enfermería, Departamento de Salud de Colectivos. Bogotá, Colombia. Email: gfuentesb@unal.edu.co Universidad Nacional de Colombia Universidad Nacional de Colombia Facultad de Enfermería Departamento de Salud de Colectivos Bogotá Colombia gfuentesb@unal.edu.co

**Keywords:** Cuidados Paliativos, Políticas Públicas de Salud, Servicios de Salud, Analgésicos Opioides, Educación, Palliative Care, Public Health Policy, Health Services, Analgesics, Opioid, Education, Cuidados Paliativos, Políticas Públicas de Saúde, Servidos de Saúde, Analgésicos Opioides, Educado

## Abstract

**Introducción:**

El desarrollo de cuidados paliativos exige la intervención de múltiples dimensiones de salud pública, incluyendo la disponibilidad de servicios de salud, medicamentos esenciales y programas educativos. En Colombia se han realizado diversos cambios en las políticas públicas para promover la atención de personas con necesidades paliativas.

**Objetivo:**

Evaluar empíricamente las políticas públicas, existentes en cuidados paliativos y sus implicaciones sobre disponibilidad de servicios, opioides y programas educativos en los años 2010 - 2019 en Colombia.

**Materiales y métodos:**

Se diseñó un estudio mixto exploratorio secuencial en tres fases: identificación de indicadores empíricos de políticas nacionales, diagnostico situacional de cuidados paliativos y evaluación cualitativa de los resultados de la implementación de políticas en siete nodos territoriales de Colombia.

**Resultados:**

Se revisaron siete normas obteniendo 12 indicadores empíricos para la evaluación, seis de ellos no contaban con fuentes de información. El diagnostico nacional evidencia un aumento gradual de servicios y consumo de opioides en los años hito del desarrollo de políticas. 44 profesionales de cuidados paliativos perciben un efecto positivo de las políticas públicas en el consumo de opioides y bajos resultados para el dominio de servicios y educación

**Conclusiones:**

Existe una relación positiva entre políticas públicas y consumo de opioides, una relación cuantitativa positiva para servicios de cuidados paliativos y una relación cuanticualitativa negativa para programas educativos, lo que denota un bajo estatus operativo de las políticas construidas para mejorar el dolor y sufrimiento asociado a la enfermedad crónica avanzada.

## Abstract (en)

**Introduction:**

Palliative care development requires the intervention of multiple dimensions of public health, including the availability of health services, essential medicines, and educational programs. In Colombia, several changes have been made in public policy to promote the care of people with palliative needs.

**Objective:**

To empirically evaluate existing public policies on palliative care and their implications for the availability of services, opioids, and educational programs during the years 2010 to 2019 in Colombia.

**Materials and methods:**

A mixed sequential exploratory study was designed in three phases: identification of empirical indicators of national policies, palliative care situational diagnosis, and qualitative assessment of the results of policy implementation in seven regional nodes in Colombia.

**Results:**

Seven standards were reviewed, yielding 12 empirical indicators for assessment, six of which had no sources of information. The national diagnosis shows a gradual increase in services and opioid use during the landmark years of policy development. Forty-four palliative care professionals perceive a positive effect of public policy on opioid use and low outcomes for service and education domains.

**Conclusions:**

There is a positive relationship between public policy and opioid use, a positive quantitative relationship with palliative care services, and a negative quantitative- qualitative relationship with educational programs. This indicates a low operational status of policies designed to alleviate the pain and suffering associated with advanced chronic diseases.

## Resumo

**Introdujo:**

O desenvolvimento dos cuidados paliativos requer a intervengo de múltiplas dimensóes da saúde pública, incluindo a disponibilidade de servidos de saúde, medicamentos essenciais e programas educativos. Na Colombia, várias mudanzas foram feitas nas políticas públicas para promover o cuidado de pessoas com necessidades paliativas.

**Objetivo:**

Avaliar empiricamente as políticas públicas existentes em cuidados paliativos e suas implicares na disponibilidade de servidos, opioides e programas educacionais nos anos 2010 - 2019 na Colombia.

**Materiais e métodos:**

Desenhou-se um estudo misto exploratório sequencial em tres fases: identificado de indicadores empíricos de políticas nacionais, diagnóstico situacional de cuidados paliativos e avaliagáo qualitativa dos resultados da implementagáo de políticas em sete nodos territoriais da Colombia.

**Resultados:**

Sete normas foram revisadas, obtendo-se 12 indicadores empíricos para avaliagáo, seis delas náo possuíam fontes de informado. O diagnóstico nacional mostra um aumento gradual nos servidos e consumo de opioides nos anos marcantes do desenvolvimento de políticas. 44 profissionais de cuidados paliativos percebem efeito positivo das políticas públicas sobre o consumo de opioides e resultados baixos para o domínio servidos e educado.

**Condusdes:**

Existe relado positiva entre políticas públicas e consumo de opioides, relado quantitativa positiva para servidos de cuidados paliativos e negativa relado quantitativo-qualitativa para programas educativos, o que denota um baixo status operacional das políticas destinadas a melhorar a dor e o sofrimento associados a doenga crónica avanzada.

## Introducción

Para 2060, se estima que 48 millones de personas morirán con graves sufrimientos relacionados con la salud, lo que representa un aumento del 87% en relación con los 26 millones de personas en 2016^1^. El 83% de estas muertes ocurrirán en personas de bajos ingresos países de ingresos medios como Colombia. Se estima que anualmente 40 millones de personas necesitan cuidados paliativos; y tan solo un 14% de las personas que necesitan asistencia paliativa la reciben. Según un informe del Observatorio Colombiano de Cuidados paliativos para el año 2016 en el país solo el 40% de la población que padece enfermedades crónicas y que afectan la calidad de vida recibieron cuidados paliativos^2^.

Para la Organización Mundial de la Salud^3^, los cuidados paliativos mejoran la calidad de vida de los pacientes y familias cuando enfrentan problemas de orden físico, psicosocial o espiritual inherentes a una enfermedad potencialmente fatal, previniendo y mejorando el sufrimiento a través de la identificación temprana, la valoración y la terapia correcta del dolor. Este cuidado comprende atender las necesidades prácticas y emocionales a la hora de sobrellevar el duelo, esta atención ofrece un sistema de apoyo para ayudar a las personas a vivir activamente hasta el día de su muerte, ya que está concebido como un derecho humano a la salud y debe ser proporcionado de manera integrada y enfocado en la persona que requiere la atención especial en sus necesidades y se debe basar en las experiencias del individuo.

Un enfoque de salud pública, como el propuesto por Stjernsward^4^, deja en claro cómo las políticas públicas son un factor determinante del progreso de los cuidados paliativos, por cuanto expresan la voluntad de un país para generar los mecanismos idóneos para el desarrollo de servicios, programas educativos y medicamentos esenciales para la atención de pacientes y familias con necesidades paliativas. De acuerdo con The 2015 Quality of Death Index Ranking palliative Care across The world^5^, el gobierno colombiano ha establecido nuevas legislaciones con el fin de mejorar el acceso de cuidados paliativos en el país, aunque esto no ha mejorado los resultados para los pacientes.

Existen múltiples metodologías para evaluar las políticas públicas, una de ellas es la evaluación empírica, definida como la forma para dar a conocer la efectividad de las políticas y medir el cumplimiento de las metas de desempeño en relación con el proceso de implementación, la perspectiva de los interesados y modificación del problema público^6^. La evaluación empírica de las Políticas Públicas comprende la observación de normas a través de un conjunto de indicadores que arrojaran datos cuantitativos, los cuales son valorados por un grupo de expertos en el tema para definir su cumplimiento, con el objetivo de mejorar la implementación de la política mediante procesos de diálogo y aprendizaje colectivo^7^.

En el caso de cuidados paliativos se han realizado múltiples evaluaciones sobre la existencia de políticas públicas como factor de desarrollo^8^^-^^10^, sin embargo, recientes estudios^11^ señalan la necesidad de evaluar el estatus operativo de las políticas en relación a modificar los factores que mejoren los resultados para el paciente. El objetivo de este estudio es evaluar de forma empírica las políticas públicas existentes en cuidados paliativos y sus efectos sobre disponibilidad de servicios, opioides y programas educativos en Colombia en el periodo 2010-2019.

## Materiales y Métodos

El presente estudio se basó en un diseño mixto exploratorio secuencial^12^ el cual se desarrolló en tres fases. En la fase I se realizó una revisión detallada de las piezas normativas reportadas en el Observatorio Colombiano de Cuidados Paliativos en el periodo 2010-2019 con el objetivo de identificar los indicadores empíricos derivados de cada norma para la evaluación de servicios, medicamentos y programas educativos. En la fase II se obtuvo una descripción cuantitativa del desarrollo de los Cuidados Paliativos a partir de los indicadores estructurados en la fase I.

Los indicadores en los que no se identificó una fuente de información para el periodo de estudio fueron excluidos del estudio. En la fase III se realizó un análisis cualitativo con representantes de diversas regiones del país y su percepción sobre el efecto de las políticas públicas en las tres áreas evaluadas. La relación de cada categoría se rateo en tres niveles, solicitando a cada participante indicar el grado en el cual los resultados de servicios, opioides y educación se relacionan con efectos normativos y/o políticos.

### Fuente de información

Fase I. Revisión detallada de las políticas públicas en Cuidados Paliativos: Para efectos de este trabajo se sigue la definición de políticas públicas como medidas regulatorias, leyes, y prioridades de gasto sobre un tema, promulgadas por una entidad gubernamental^13^, por lo que se incluyeron todas las piezas normativas, sin distinción de su jerarquía (Ley, decreto, resolución, etc.) relacionadas con cuidados paliativos y el final de la vida. La información fue organizada en una ficha de captura para el respectivo análisis.

Fase II. Descripción cuantitativa del desarrollo de los cuidados paliativos: Para servicios de cuidados paliativos, se utilizó el registro especial de prestadores de salud (REPS) del Ministerio de Salud y Protección Social de Colombia^14^ y el Observatorio Colombiano de Cuidados Paliativos^2^. Para medicamentos opioides los datos provienen del Fondo nacional de estupefacientes y el Walther Center in Global Palliative Care de la Universidad de Indiana^15^ que realiza un monitoreo global del consumo de medicamentos opioides. Para educación, se identificaron como fuentes de información el Sistema Nacional de Información de la Educación Superior (SNIES), el Observatorio Colombiano de Cuidados Paliativos^2^ y los sitios web de las instituciones de educación superior del país. (IESS), así como aseguradores y prestadores de servicios de salud.

Fase III. Descripción cualitativa de los efectos de las políticas sobre servicios, opioides y programas educativos: Para garantizar la representatividad nacional, el grupo de investigadores utilizó una clasificación por nodos desarrollada por León y colaboradores^16^. Para la recolección de información se desarrolló una entrevista estructurada a partir de los hallazgos de la fase II, en la cual se les pide a los participantes indicar cómo se percibe los efectos de la política pública en el desarrollo de servicios, medicamentos y programas educativos para cuidados paliativos en Colombia. En la Tabla 1, se presenta las regiones que conforman cada nodo participante.

**Tabla 1:** Nodos territoriales del estudio

Nodo	Departamento
Amazonia	Putumayo, Amazonas, Caquetá, Guaviare, Vaupés y Guainía.
Orinoquía	Meta, Vichada, Casanare, Arauca y Cundinamarca.
Nororiente	Boyacá, Santander, Norte de Santander y Cesar.
Pacífico	Nariño, Cauca, Valle del Cauca y Chocó.
Centro	Antioquia, Caldas, Quindío, Risaralda, Tolima y Huila.
Caribe	La Guajira, Magdalena, Atlántico, Bolívar, Sucre, Córdoba, San Andrés.
Bogotá	Bogotá

Este estudio fue aprobado por el comité institucional de ética en investigación de la Universidad El Bosque, recibiendo aval para su ejecución.

### Análisis de datos

La base de datos fue almacenada en Mendeley Data^17^. En la primera fase se realizó una síntesis estructurada de las piezas normativas y se definieron indicadores para monitorear su desempeño, definiciones operativas y fuentes de información. En la fase II se agrupan los resultados para los años donde se desarrollan un mayor número de piezas normativas, evaluando la tendencia de los indicadores identificados. Para la fase III, un análisis cualitativo descriptivo examina la relación de las políticas con respecto a cada una de las áreas evaluadas.

## Resultados

### Indicadores empíricos para la evaluación del estatus operativo de las políticas

Se analizaron siete normas relacionadas con cuidados paliativos y del final de la vida obteniendo 12 indicadores empíricos para la evaluación del desarrollo. En la Tabla 2 se presentan los indicadores empíricos generados a partir de la revisión normativa y se incluye la fuente de información para la obtención de datos del periodo de estudio. Seis indicadores no cuentan con fuente de información o las mismas no permiten la evaluación de los indicadores en la totalidad de años de este estudio.

**Tabla 2:** Indicadores empíricos para le evaluación de la política pública

Dominio	Indicador empírico	Definición operative	Norma de la que procede	Fuente de información
Servicios	Número de servicios de cuidado paliativo habilitado en el país	Número de servicios habilitados en el país para la provisión de Dolor y Cuidados Paliativos.	Resolución 1416 de 2018.	Registro especial de prestadores de servicios de salud
	Número de entidades administradoras de planes en beneficios en salud que ofrecen a sus afiliados la prestación del servicio de cuidado paliativo	Entidades administradoras de planes de beneficios en salud de servicios que ofertan cuidados paliativos en Colombia	Ley 1733 del 2014.	No hay forma de obtener información en una línea de tiempo
	Número de casos de muertes por condiciones plausibles del cuidado paliativo	Muertes de pacientes crónicos que necesitaron cuidados paliativos.	Ley 1733 de 2014.	No hay forma de obtener información en una línea de tiempo
	Existencia de un protocolo o guía de práctica clínica para el manejo de pacientes en cuidados paliativos	Existe una guía nacional para el manejo de pacientes con necesidades paliativas	Resolución 1441 de 2016	Ministerio de salud y Protección Social
Opioides	Total de opioides consumidos al año/ el total de opioides requeridos legalmente al año	Consumo de opioides anual reportado- excluyendo Metadona- en Morfina equivalente (per cápita)	Resolución 1478 de 2006.	Walther Center in Global Palliative Care de la Universidad de Indiana
	Total de centros de atención primaria con disponibilidad de morfina oral / en total de centros de atención primaria	Proporción centros de atención primaria que cuentan con disponibilidad de morfina oral de liberación inmediata (líquido o comprimido).	Ley 1753 de 2015.	No hay forma de obtener información en una línea de tiempo
	Número de establecimientos autorizados para la distribución y venta de medicamentos opioides / el total de establecimientos que venden y distribuyen opioides.	Proporción de establecimientos autorizados para la distribución y venta de medicamentos opioides.	Circular 22 de 2016.	No hay forma de obtener información en una línea de tiempo
	Número de personas que acceden a medicamentos no incluidos en el plan de beneficios/ total de personas que requieren medicamentos no incluidos en el plan de beneficios	Porcentaje de personas que acceden a medicamentos no incluidos en el plan de beneficios para el control de dolor y otros síntomas productores de sufrimiento.	Circular 22 de 2016.	No hay forma de obtener información en ninguna línea de tiempo
	Número de centros de dispensación de medicamentos opioides con disponibilidad 24/7	Servicios farmacéuticos qué operan de forma permanente para garantizar la entrega continua de medicamentos para aliviar el dolor.	Ley 1733 de 2014.	No hay forma de obtener información en una línea de tiempo
Educación	Número de escuelas de medicina con inclusión de cuidados paliativos en la formación de pregrado	Programas de medicina que incluyen en su plan de estudios la asignatura de cuidados paliativos	Resolución 063, Asamblea mundial de salud.	Sitio web IESS con programas de medicina
	Número de escuelas de enfermería con inclusión de cuidados paliativos en pregrado	Programas de enfermería que incluyen en su plan de estudio cuidados paliativos	Resolución 063, Asamblea mundial de salud.	Sitio web IESS con programas de enfermería
	Número de programas de educación continuada en cuidados paliativos	Programas de educación continuada en cuidados paliativos ofertados por instituciones de educación superior, aseguradores o prestadores de servicios de salud	Ley 1733 de 2014.	Sitio web IESS aseguradores y prestadores de servicios de salud

Las fuentes de información que se construyen en este estudio tienen un carácter oficial en tres indicadores y un carácter privado en los restantes. Llama la atención que el monitoreo de consumo de opioides no está disponible con recursos nacionales, como si lo hacen observatorios internacionales. El Observatorio Colombiano de Cuidados Paliativos, presenta la información de algunos de los indicadores evaluados, sin embargo, se privilegian datos primarios en este estudio.

### Descripción cuantitativa del desarrollo de los cuidados paliativos

Al utilizar los seis indicadores se evidencia el comportamiento que tienen los servicios, opioides y programas educativos en tres momentos del periodo evaluado (2010, 2014, 2019), los cuales se consideran hitos en el desarrollo normativo, por tener una mayor actividad política o legislativa, ver Tabla 3.

**Tabla 3:** Diagnostico situacional de Cuidados Paliativos en años hito de actividad política

Servicios	Número de servicios de cuidado	63	102	454
	paliativo habilitado en el país			
	Existencia de un protocolo o guía de práctica clínica para el manejo de pacientes en cuidados paliativos	NO	NO	SI
Opioides	Total de opioides consumidos en equivalentes de Morfina	8mg	14mg	17mg
Educación	Número de escuelas de medicina con inclusión de cuidados paliativos en la formación de pregrado	1	2	4
	Número de escuelas de enfermería con inclusión de cuidados paliativos en pregrado	1	2	6
	Número de programas de educación continuada en cuidados paliativos	0	3	5

En 2010 fue expedida la primera reglamentación de cuidados paliativos en Colombia y se contaba con 63 servicios, en 2014 se expide la Ley Nacional de Cuidados Paliativos, aumentando a 102, en 2019 se llega a 454 servicios. Este crecimiento es heterogéneo en diferentes regiones del país, evidenciando territorios en donde se presentan un número limitado de servicios versus ciudades con mayor densidad poblacional. Seis departamentos, San Andrés y Providencia, Putumayo, Guainía, Vaupés y Vichada, Amazonas, no cuentan con ningún servicio, aun cuando la Ley tiene un alcance nacional.La Ley nacional de Cuidados Paliativos señala la necesidad de desarrollar estrategias para el consumo de opioides. El análisis cuantitativo evidencia un aumento en los mg/percapita de morfina consumidos (indicador internacional para el consumo de opioides fuertes). Se evidencia un desarrollo inequitativo en regiones como Quindío, Risaralda, Atlántico y Antioquia versus territorios como Amazonas, Arauca, Cesar, Chocó, Guainía, Guaviare, donde el consumo sigue siendo extremadamente bajo.

La formación para profesionales ha venido aumentando, solo hasta el 2014 la Ley nacional señala la necesidad de desarrollar programas de formación continuada y en el 2016 la expedición de los decretos reglamentarios de la Ley 1733 de 2014, incluyen la formación en Cuidados Paliativos como parte de los estándares del sistema nacional de habilitación en salud. Sin embargo, el número de programas de pregrado de medicina y enfermería que incluyen contenidos de cuidados paliativos es baja en comparación con el total nacional de facultades.

### Descripción cualitativa del desarrollo de los Cuidados Paliativos

44 profesionales que laboran en cuidados paliativos participaron en esta fase, los datos cualitativos fueron analizados y categorizados en los tres dominios de estudio. La Tabla 4 presenta una relación de los resultados entre políticas públicas, servicios, opioides y educación, indicando los factores más frecuentes en la evaluación cualitativa.

**Tabla 4:** Relación percibida entre políticas públicas y servicios, opioides y programas educativos.

Nodo	Servicios	RPP	Opioides	RPP	Educación	RPP
Amazonía	Barreras de acceso	Baja	Barreras de disponibilidad.	Baja	Diversificar las modalidades de educación y los beneficiarios	Baja
Bogotá D.C	Normas abstractas	Baja	Consumo adecuado	Alto	Limitar competencias	Media
Caribe	Barreras de acceso	Baja	Consumo adecuado	Alto	Diversificar las modalidades de educación y los beneficiarios	Baja
Centro	Normas abstractas	Baja	Consumo adecuado	Alto	Ampliar formación a otras disciplinas	Media
Nororiente	Barreras de acceso	Baja	Consumo adecuado	Alto	Diversificar las modalidades de educación y los beneficiarios	Baja
Orinoquía	Barreras de acceso	Baja	Barreras de disponibilidad	Medio	Diversificar las modalidades de educación y los beneficiarios	Baja
Pacifico	Barreras de acceso	Baja	Consumo adecuado	Alto	Diversificar las modalidades de educación y los beneficiarios.	Baja

*RPP: Relación con Políticas Publicas*

### Servicios

Desde la perspectiva de los participantes existen barreras de acceso, la normatividad tiene claro el componente de cuidados paliativos, pero no existe una directriz clara, dada la ausencia de un plan operativo para garantizar la atención. Los aseguradores continúan restringiendo los servicios, en un sistema financiero que no comprende ni permite priorizar los Cuidados Paliativos. Los problemas de acceso son descritos especialmente en poblaciones de menos recursos. Se requieren más acciones de inspección, vigilancia y control sobre las aseguradoras para garantizar los derechos. Aunque se ha avanzado, aún falta una mayor divulgación y consolidación de los servicios.

### Consumo de opioides

El consumo per cápita ha mejorado gracias al esfuerzo normativo de asegurar la disponibilidad de medicinas para el alivio del dolor. En Bogotá, el nodo Pacífico, Nororiental, Caribe y Centro, las normas y programas favorecen en un alto grado el acceso. En los nodos de Orinoquía y Amazonía, se evalúa entre medio y bajo, lo que afecta la calidad de los servicios brindados y la satisfacción de los pacientes. En Bogotá se reconoce que el consumo ha mejorado a expensas del trabajo gubernamental, pero los participantes rescatan la labor de las asociaciones científicas y la academia quienes con su gestión favorecen la disponibilidad de opioides. Los participantes señalan que el aumento per cápita de consumo de opioides en Colombia está dado en las dos o tres ciudades más importantes del país. En la región caribe los entrevistados exponen el consumo de opioides como muy bajo, en parte por la opiofobia y la falta de experticia de los profesionales, lo que deriva en la no prescripción de estos fármacos a pesar de los esfuerzos normativos por mejorar el consumo racional de opioides.

### Educación

Los esfuerzos políticos y legislativos no tienen unas directrices claras, por lo que gran parte de iniciativas y gestión se ha hecho desde la academia y asociaciones. Siguen siendo pocos los programas de pregrado y posgrados disponibles para los profesionales, la gran mayoría se concentran en dos ciudades. En los nodos de Amazonía y Orinoquía, los participantes señalan que se debe fortalecer a los promotores de salud, técnicos auxiliares, voluntarios, implementando nuevas modalidades de capacitación. Muchos pacientes y familias desconocen el propósito de los cuidados paliativos, por lo que se considera ofrecer capacitación para mejorar la cobertura de los cuidados paliativos.

En los nodos Centro, Caribe y Pacífico existe la necesidad de ampliar la formación, actualmente solo se contempla el entrenamiento de profesionales de enfermería y medicina, siendo necesario incluir a rehabilitadores, profesionales de apoyo psicosocial y trabajadores espirituales, los cuales son invisibles para los reguladores. Se identifica que no se cuenta con instituciones educativas ni personal capacitado, por lo que en nodos como Bogotá, Nororiente y Pacífico existe la necesidad de formar profesionales entrenados, en el componente primario de atención. En los nodos de Bogotá y Centro se debe estructurar un esquema normativo que delimite competencias profesionales, dado que advierten un desarrollo heterogéneo de programas que no garantizan una atención segura del paciente

## Discusión

Las políticas públicas han sido consideradas desde hace más de 15 años la base sobre la cual se desarrollan los cuidados paliativos en el mundo^4^. 12 indicadores de evaluación empírica de los cuidados paliativos fueron identificados, de los cuales 6 no cuentan con fuentes de información que permitan monitorear el cumplimiento de las acciones establecidas en las políticas públicas. Las políticas públicas pueden construirse de forma abstracta sin generar acciones específicas que mejoren los resultados para las personas que necesitan acceder a cuidados paliativos, lo cual se evidencia en la dificultad para monitorear el desempeño de la reglamentación establecida^6^^,^^7^^,^^11^.

Trabajos como el de Arias-Casáis y colaboradores, establecen un conjunto de indicadores que permiten monitorear el desarrollo de cuidados paliativos y con base en ellos se ha calificado a Colombia como un país de provisión generalizada^12^^,^^13^, sin embargo, cuando se examina el efecto de las políticas se evidencia que, para el caso de servicios, existe un aumento de los servicios en los años donde existen hitos de políticas públicas. De igual forma, el consumo de opioides aumenta con relación a la expedición de normas que reglamentan la disponibilidad y acceso a estos medicamentos^16^, no así los programas educativos en esta materia.

Una perspectiva cualitativa del problema deja ver como existe una marcada tendencia a considerar una baja relación entre las políticas públicas y los efectos en servicios y educación^18^. Los participantes consideran en los siete nodos evaluados como el consumo de opioides ha mejorado de manera significativa por la acción de las políticas establecidas en esta materia. Salvo en los nodos de Amazonas y Orinoquía, la relación de opioides y Políticas Públicas es considerada alta. Estos hallazgos difieren de los estudios publicados por León y Colaboradores^19^^-^^21^, en donde de describe de forma general una limitación del consumo de opioides a partir de las políticas y la regulación restrictiva.

En particular, en cuidados paliativos se identifica un sustancial aumento de las normas expedidas y la actividad legislativa relacionada, sin que ello conduzca a un estatus operativo de las políticas, por lo que se debe considerar la expedición de decretos operativos y locales que respondan a las condiciones de salud de la población de los diferentes territorios del país. Se debe señalar como limitación del estudio la necesidad de incluir en el análisis la relación entre políticas públicas y determinantes sociales en salud, lo cual se considera un área futura de investigación para comprender con mayor profundidad el desarrollo equitativo de cuidados paliativos en Colombia.

## Conclusión

Existe un desarrollo heterogéneo de los resultados entre políticas públicas y cuidados paliativos, pese a que en la práctica los componentes evaluados se combinan en busca de mejorar los resultados para los pacientes y sus familias. Este trabajo encontró a partir de seis indicadores empíricos de las políticas públicas de cuidados paliativos una relación cuanticualitativa positiva para medicamentos opioides, una relación cuantitativa positiva para servicios y una relación cuanticualitativa negativa para programas educativos.
